# Perioperative Management of a Patient With Fontan Circulation Presenting for Laparoscopic Cholecystectomy: A Case Report

**DOI:** 10.7759/cureus.115741

**Published:** 2026-09-03

**Authors:** Nathan Hyre, Jason Greib, Selena Rashid, Ibtesam Hilmi

**Affiliations:** 1 Department of Anesthesiology and Perioperative Medicine, University of Pittsburgh Medical Center (UPMC) Medical Education, Pittsburgh, USA

**Keywords:** adult congenital heart disease, cholecystectomy, fontan circulation, laparoscopic surgery, perioperative management

## Abstract

We present the perioperative management of a 22-year-old female patient with congenital heart disease and Fontan circulation presenting for laparoscopic cholecystectomy. Careful preoperative review of recent cardiac testing and assessment of the patient’s physical status allowed for an individualized anesthetic approach. Perioperative management began in the preoperative holding area with preload optimization. Intraoperative management focused on supporting flow through the Fontan circulation through positioning adjustments, ventilatory management, and vasopressor support. Collaboration with the surgical team to minimize insufflation pressures and head-up positioning promoted adequate venous return. Minimizing peak airway pressures and maintaining a mild respiratory alkalosis mitigated the pulmonary vasoconstricting effects of hypercapnia. Additionally, vasopressin was administered via intermittent boluses and continuous infusion to maintain adequate systemic vascular resistance while minimizing pulmonary vasoconstrictive effects. The perioperative course was uncomplicated, and the patient was discharged home after overnight observation. This case adds to existing literature on anesthetic management of adults with Fontan circulation presenting for noncardiac surgery and presents a unique combination of vasopressor choice and minimally invasive monitoring when compared to prior case reports. The case also highlights the importance of interdisciplinary collaboration and individualized anesthetic management for this patient population.

## Introduction

The Fontan procedure remains the final palliative surgery for patients with congenital heart defects when two-ventricle circulation is not feasible. Advancements in surgical technique and perioperative management of patients post-Fontan have led to long-term improvement in survival. For patients operated on today, 30-year survival is ~80% [1], and the estimated global population of patients living with Fontan palliation has grown from 50,000 to 70,000 [2]. Consequently, the number of adult Fontan patients requiring anesthesia for noncardiac procedures continues to increase.

Patients with single ventricle physiology are at high risk of adverse perioperative events when undergoing noncardiac surgery [3]. The sensitivity of this patient population to changes in preload, pulmonary vascular resistance (PVR), and single-ventricle function presents unique challenges for the anesthesia provider. Laparoscopic cholecystectomies, which are typically performed in a head-up position (reverse Trendelenburg) with CO_2_ peritoneal insufflation and positive-pressure ventilation, entail additional intraoperative hemodynamic considerations.

Several review articles have been published regarding the general perioperative management of patients with Fontan physiology undergoing noncardiac surgery [4-6]. However, the wide variation in complex congenital anatomy, single-ventricle function, and the unique challenges of laparoscopic surgery warrant a patient-specific anesthesia plan. In this report, we describe the successful anesthetic management of an adult female patient with Fontan circulation who presented via same-day surgery to a tertiary care center for a laparoscopic cholecystectomy.

## Case presentation

The patient was a 22-year-old obese female (height 160 cm, weight 92 kg, and BMI 36) with symptomatic cholelithiasis presenting for laparoscopic cholecystectomy. She was diagnosed with pulmonary valve stenosis and a double-inlet left ventricle at birth. The surgical management of her congenital heart disease (CHD) included a modified Blalock-Taussig-Thomas Shunt at three days of life to create a temporary connection between the subclavian and pulmonary arteries to increase pulmonary blood flow and a bidirectional Glenn procedure at nine months of age to connect the superior vena cava to the right pulmonary artery.

She then underwent an extracardiac fenestrated 20-mm Fontan conduit at four years of age. At 15 years of age, fenestration closure was performed due to hypoxia and poor functional capacity. The systemic ventricle in this patient was a double-inlet morphologic left ventricle (Figure 1), and a recent transthoracic echocardiogram demonstrated patent Glenn and Fontan conduits (Figure 2) with normal diastolic and systolic left ventricular function (ejection fraction of 55%).

**Figure 1:**
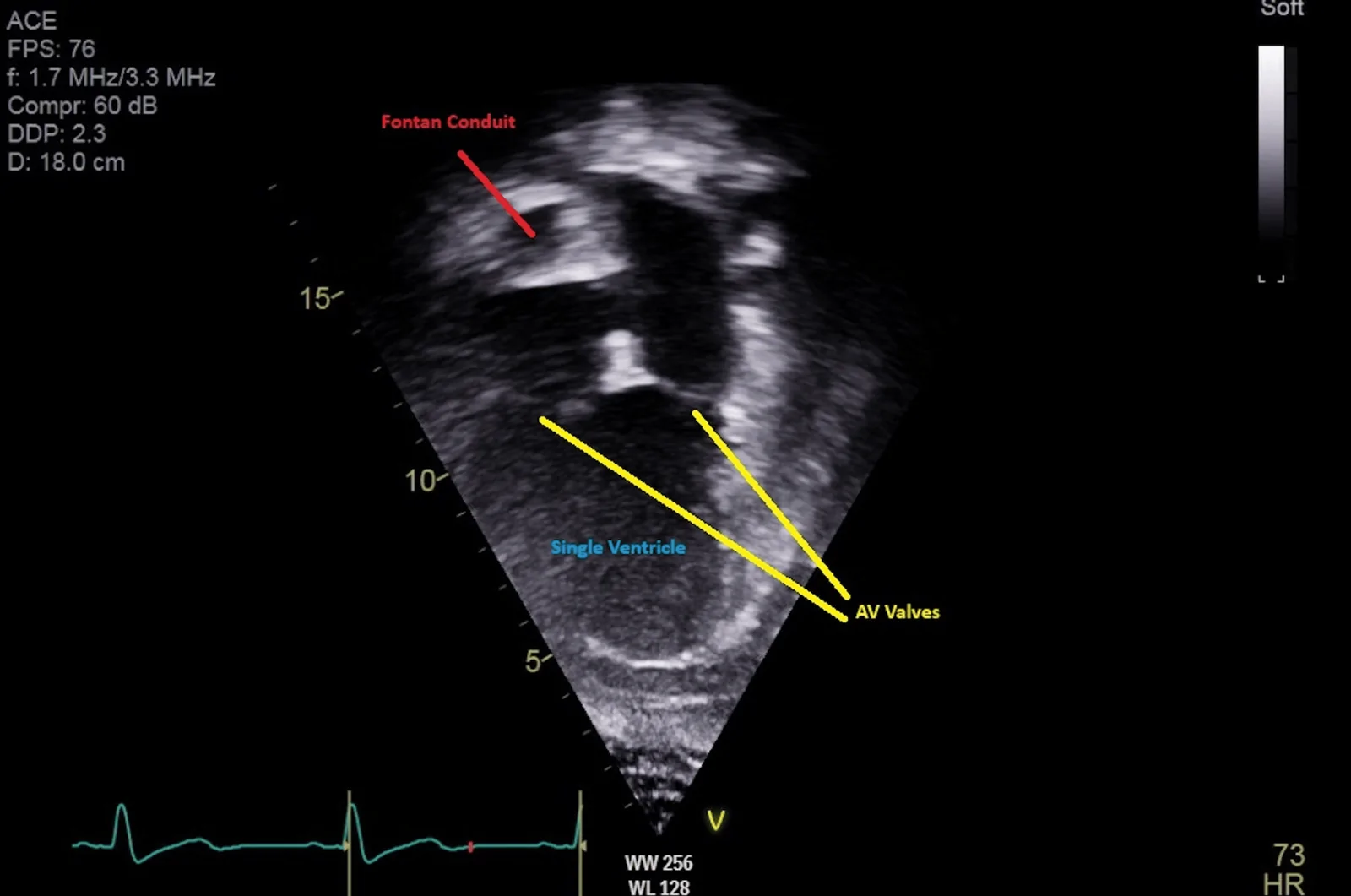
Apical four-chamber view of the double inlet left ventricle and Fontan conduit AV: atrioventricular

**Figure 2:**
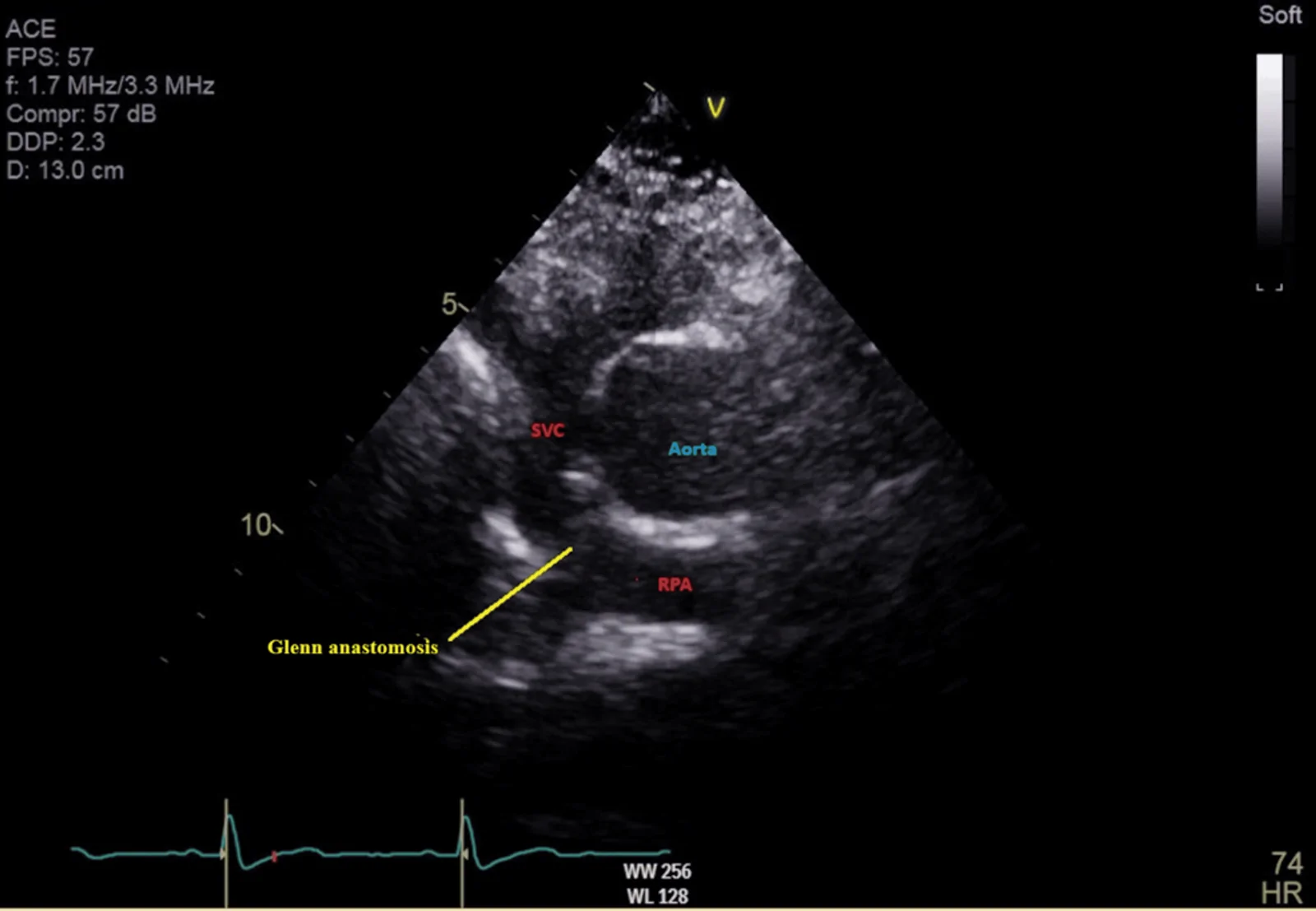
Suprasternal short-axis view of patent Glenn anastomosis RPA: right pulmonary artery; SVC: superior vena cava

Additional medical history included sleep apnea diagnosed via home sleep testing, postoperative nausea and vomiting (PONV), and severe generalized anxiety disorder related to prior healthcare experiences. The patient had no history of arrhythmias (with a recent normal 12-day Holter monitor), and she was not taking anticoagulation. Preoperative laboratory testing, including electrolytes and liver function tests, was within normal limits. On the morning of surgery, she denied chest pain, dyspnea, or orthopnea, and she reported adequate functional capacity (more than four metabolic equivalents). Physical exam revealed no signs of chronic hypoxia, volume overload, or venous congestion. Baseline vital signs in preoperative holding were temperature 36.6°C, heart rate 77 beats/minute with normal sinus rhythm, blood pressure 147/97, and oxygen saturation of 96% on room air. She was classified as an American Society of Anesthesiologists Physical Status III due to her complex CHD and single-ventricle physiology.

Preoperative management included a 1-L intravenous (IV) fluid bolus of Plasma-Lyte A to address fasting deficits, a scopolamine patch for PONV prophylaxis, and 2 mg of IV midazolam for anxiolysis. In the operating room, the patient was placed in standard supine positioning, and a radial artery line was placed under ultrasound guidance prior to induction. General anesthesia was induced using 100 mg of IV 2% lidocaine, 100 mcg of fentanyl, 30 mg of etomidate, and 200 mg of succinylcholine. Cricoid pressure was applied to facilitate an uneventful rapid sequence intubation. Sevoflurane in air and oxygen was used to maintain general anesthesia, with inhaled nitric oxide available if needed. Given the patient’s history of PONV, dexamethasone was administered after induction, and a low-dose propofol infusion (20-50 mcg/kg/minute) was continued intraoperatively.

Using pressure-controlled ventilation with a preset positive end-expiratory pressure (PEEP) of 3 cmH_2_O, an initial peak inspiratory pressure of 18 cmH_2_O was required to deliver a tidal volume of 7 mL/kg of ideal body weight. The patient was hyperventilated with a respiratory rate of 16-20 breaths/minute to achieve mild respiratory alkalosis as confirmed by arterial blood gas and end-tidal CO_2_ monitoring. The patient was incrementally placed in reverse Trendelenburg while monitoring the blood pressure response. Similarly, CO_2_ peritoneal insufflation was performed incrementally to an intra-abdominal pressure (IAP) of ~10 mmHg while communicating with the surgical team. Of note, the patient experienced mild hypotension (~70/40, with mean arterial pressures (MAPs) in the 50s) that was unresponsive to vasopressors during positioning, which resolved with bed leveling and temporary desufflation.

Intraoperative hypotension was managed with a combination of positional changes, IV fluids as guided by pulse pressure variation, and vasopressors. IV vasopressin as a bolus (one to three units) and infusion (0.02 units/minute) was the sole vasoactive agent used intraoperatively. The procedure proceeded without complication, with a total operating time of 87 minutes and a total time under general anesthesia of 138 minutes. The patient was extubated in the operating room and transferred to the postanesthesia care unit, where her post-op course was uncomplicated. Out of an abundance of caution, the patient was monitored overnight and discharged home on post-op day 1. During outpatient follow-up two weeks after discharge, she reported resolution of preoperative symptoms, adequate oral intake, and return of normal bowel function with no apparent complications.

## Discussion

Since the initial Fontan procedure in 1968 on a patient with tricuspid atresia [7], the prevalence of adults living with Fontan circulation has continued to rise [2], and this patient population often requires noncardiac surgery. While long-term outcomes of adult Fontan patients are encouraging [8], the variation in complex anatomy, functional status, and possible co-morbid conditions [9] creates significant heterogeneity within this population. Understanding this variety and creating an individualized anesthetic plan is essential for safe perioperative management of adult Fontan patients.

Flow through the pulmonary vasculature, which depends on central venous pressure (CVP), PVR, and ventricular diastolic pressure, is the primary physiologic mechanism supporting Fontan circulation [10]. In this case, we implemented a multifaceted approach to maintain this physiology. Administering a preoperative fluid bolus allowed us to optimize preload and address the fasting fluid deficit. Additionally, placing a preinduction arterial line and inducing general anesthesia with a stable cardiovascular agent such as etomidate [11] minimized peri-induction hemodynamic changes.

Intraoperative management focused on minimizing changes in PVR and counteracting the physiologic challenges of laparoscopic surgery. Reverse Trendelenburg positioning and increased IAP during laparoscopic surgery can impair venous return and increase PVR via CO_2 _absorption and hypoventilation [12,13]. However, maintaining low IAP may minimize these cardiovascular effects [14]. Several case reports have documented successful laparoscopic surgeries in patients with Fontan circulation by maintaining IAP less than 12 mmHg [15,16]. Through collaboration with the surgical team, we optimized positioning without steep reverse Trendelenburg or high IAP.

From a ventilatory standpoint, maintaining a mild respiratory alkalosis further mitigated the effects of partial pressure of carbon dioxide (PaCO_2_) on PVR. Serial titration of minute ventilation to compensate for rising PaCO_2_ with increasing insufflation time is an important consideration. Given the anticipated short procedural duration, we felt the benefits of minimizing airway pressures via a low PEEP strategy (<5 cmH_2_O) outweighed the risks of intraoperative atelectasis.

Using intermittent boluses and a continuous infusion of vasopressin allowed us to address the vasodilatory effects of general anesthetics to maintain adequate MAPs with minimal increases in PVR. Vasopressin has advantages over norepinephrine and other alpha agonists because it has minimal effect on the PVR. Milrinone, which has been successfully administered intratracheally [15] and as an IV infusion [16] to support Fontan circulation due to its inodilator properties, was available but ultimately unnecessary given the patient’s adequate single ventricular function. Similarly, inhaled nitric oxide was available if needed as a pulmonary vasodilator but deferred, as it was not needed. Finally, we opted to forego additional invasive CVP or TEE monitoring given the short procedural duration and the anticipated patient discharge home.

## Conclusions

We report the successful perioperative management of an adult patient with Fontan circulation presenting for laparoscopic cholecystectomy. This report reiterates the importance of communication with the surgical team during positioning and insufflation. Additionally, individualized preoperative assessment and planning combined with careful intraoperative ventilatory and vasopressor management allowed for maintenance of baseline patient physiology. The combination of vasopressin as the primary vasopressor with minimal invasive monitoring distinguishes this case from existing reports. Given that this manuscript discusses a single-patient case report, there is limited ability to draw generalizable conclusions about the patient population. However, this report may help familiarize anesthesia providers with the complex physiology of patients with Fontan circulation and provide an example of perioperative clinical decision making.
